# Dynamics of centriole amplification in centrosome-depleted brain multiciliated progenitors

**DOI:** 10.1038/s41598-019-49416-2

**Published:** 2019-09-10

**Authors:** Olivier Mercey, Adel Al Jord, Philippe Rostaing, Alexia Mahuzier, Aurélien Fortoul, Amélie-Rose Boudjema, Marion Faucourt, Nathalie Spassky, Alice Meunier

**Affiliations:** 1 Institut de Biologie de l’École Normale Supérieure (IBENS), Paris Sciences et Lettres (PSL) Research University, Paris, F-75005 France; 2 CNRS, UMR 8197, Paris, F-75005 France; 3 INSERM, U1024, Paris, F-75005 France; 4 Center for Interdisciplinary Research in Biology (CIRB), Collège de France, CNRS 7241 INSERM U1050, PSL Research University, Paris, 75005 France

**Keywords:** Cellular imaging, Organelles

## Abstract

Reproductive and respiratory organs, along with brain ventricles, are lined by multiciliated epithelial cells (MCC) that generate cilia-powered fluid flows. MCC hijack the centrosome duplication pathway to form hundreds of centrioles and nucleate motile cilia. In these cells, the large majority of procentrioles are formed associated with partially characterized organelles called deuterosomes. We recently challenged the paradigm that deuterosomes and procentrioles are formed *de novo* by providing data, in brain MCC, suggesting that they are nucleated from the pre-existing centrosomal younger centriole. However, the origin of deuterosomes and procentrioles is still under debate. Here, we further question centrosome importance for deuterosome and centriole amplification. First, we provide additional data confirming that centriole amplification occurs sequentially from the centrosomal region, and that the first procentriole-loaded deuterosomes are associated with the daughter centriole or in the centrosomal centriole vicinity. Then, to further test the requirement of the centrosome in deuterosome and centriole formation, we depleted centrosomal centrioles using a Plk4 inhibitor. We reveal unexpected limited consequences in deuterosome/centriole number in absence of centrosomal centrioles. Notably, in absence of the daughter centriole only, deuterosomes are not seen associated with the mother centriole. In absence of both centrosomal centrioles, procentrioles are still amplified sequentially and with no apparent structural defects. They seem to arise from a focal region, characterized by microtubule convergence and pericentriolar material (PCM) assembly. The relevance of deuterosome association with the daughter centriole as well as the role of the PCM in the focal and sequential genesis of centrioles in absence of centrosomal centrioles are discussed.

## Introduction

Multiciliated cells (MCC) grow up to several hundred of motile cilia to generate fluid flow necessary for proper respiratory, reproductive, and brain functions^1^. These cilia are nucleated by centriole-derived basal bodies docked at the plasma membrane. Multiciliated precursors, containing only the two centrioles of their centrosome, must therefore amplify up to 300 centrioles to allow cilia nucleation. This amplification occurs thanks to intermediate organelles, the deuterosomes, which are composed of centriole-related elements^2,3^. Deuterosomes support massive centriole production by a molecular cascade that mimics the centrosome duplication program^3–5^. Because two centrosomal centrioles seemed insufficient to scaffold the formation of tens of centrioles, massive centriole production through deuterosome structures was proposed to arise independently from the centrosome in MCC^6^. Challenging this postulate, an electron microscopy study showing association of deuterosomes with centrosomal centrioles in chick trachea proposed that the “procentriole clusters may form initially in close association with the diplosomal centrioles”^7^. More recently, we highlighted an atypical asymmetry between the mother and daughter centriole of the centrosome during cultured brain MCC differentiation, where deuterosomes are seen associated with the daughter centriole by electron microscopy^8^. Centriole amplification dynamics revealed by Cen2GFP live imaging together with centriole duplication players (Cep152, Plk4, Sas6) and Deup1 accumulation at the daughter centriole suggested that most procentrioles were amplified sequentially from the young centrosomal centriole through deuterosome formation. It also suggested the existence of a local micro-environment conductive to the formation of these auxiliary centrosome structures^8^. In mouse tracheal epithelial cell cultures, the transcription factor E2F4 was shown to accumulate in the centrosomal region during centriole amplification, and to be involved in deuterosome formation^9^. More recently, pericentriolar material proteins PCNT and γ-Tubulin were shown to associate with the deuterosomes^10^. Here using new tools available to study centriole amplification in MCC –a home-made Deup1 antibody and the Plk4 inhibitor centrinone- we further investigate the relationship between the centrosome organelle and the dynamics of centriole amplification by (i) characterizing the centrosome behavior during centriole amplification and (ii) assessing the dynamics of amplification in cells depleted from one or both centrosomal centrioles.

## Results

### Centriole amplification proceeds sequentially and begins from the centrosomal region

**Figure 1:**
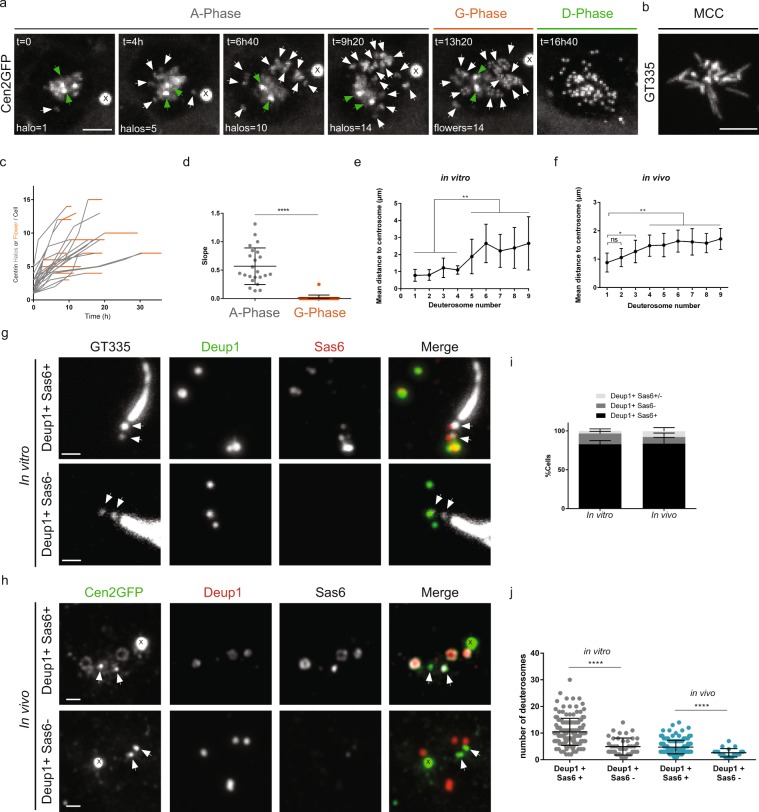
Centriole amplification proceeds sequentially and arises from the centrosomal region. (**a**) Time lapse sequences of a Cen2GFP ependymal progenitor undergoing the different stages (A-Phase, G-Phase and D-Phase) of centriole amplification. Early A-Phase is characterized by Cen2GFP cloud surrounding centrosomal centrioles and the first visible halos in the nearby cytoplasm. As amplification progresses, the number of halos increases and they localize throughout the cytoplasm. In G-Phase, the final number of halos is reached, Cen2GFP halos transform into flowers where procentrioles are becoming visible. In D-Phase, procentrioles individualize. White arrows indicate centrin halos or flowers. Green arrows indicate centrosomal centrioles. (**b**) Cilia immunostaining with GT335 antibody of a Cen2GFP ependymal MCC at the end of a time lapse experiment. (**c**) Number of centrin halos (Gray) or flowers (Orange) during time lapse experiments in Cen2GFP ependymal progenitors (Δt = 40 min, n = 23 cells). Halos or flowers are sometimes masked because of their backward movements toward the centrosomal region. Consequently, they have been counted during expansion phases when each single structure is clearly visible by 3D monitoring. (**d**) Trend line slopes corresponding to each cell observed in **c** in A-Phase and G-phase. (**e**,**f**) Mean distances of deuterosomes to the centrosome depending on the number of deuterosomes in the cell *in vitro* (**e**, n = 67 cells), and *in vivo* (**f**, n = 134 cells). Deuterosomes were counted when positive for Deup1 and Sas6 stainings. (**g**,**h**) Immunostaining of cells in A-phase with loaded (Deup1+/Sas6+) or unloaded (Deup1+/Sas6−) deuterosomes, *in vitro* (**g**), and *in vivo* (**h**). Arrows indicate centrosomal centrioles. (**i**) Quantification of cells with loaded (Deup1+/Sas6+), partially loaded (Deup1+/Sas6+/−) or unloaded deuterosomes (Deup1+/Sas6−) *in vitro* (n = 128 cells) and *in vivo* (n = 279 cells). (**j**) Quantification of the number of deuterosomes depending on the loading status of deuterosomes *in vitro* and *in vivo* (n = 196 cells *in vitro*, n = 165 cells *in vivo*). « X » indicates GFP aggregates. Scale bars: (**a**,**b**) 5 µm; (**g**,**h**) 1 µm.

We have recently shown that centriole amplification in mouse ependymal progenitor cells is marked by 3 phases^8^. The amplification A-phase, where procentrioles form on centrosome and deuterosome platforms, the growth G-phase, during which all procentrioles elongate and mature synchronously, and the disengagement D-phase, during which maturing centrioles are released from their growing platforms to dock at the apical plasma membrane, become basal bodies, and nucleate motile cilia. In order to precise early A-phase dynamics, we used our *in vitro* assay where the differentiation of mouse Centrin 2 GFP-tagged (Cen2GFP) ependymal cells allows the monitoring of centriole amplification dynamics (Fig. 1a,b, Supplementary Movie 1)^8,11^. We first focused on the formation of immature procentrioles around deuterosomes (taking the form of Cen2GFP “halos”) during the A-phase and their subsequent maturation, visible by the transformation of Cen2GFP halos into “flowers” during the G-phase. We quantified that (i) the number of Cen2GFP halos increased over time in all the cells (Fig. 1a,c; grey), confirming, as previously stated^8^, that procentrioles are formed sequentially in the brain MCC progenitor, and (ii) the maturation of procentrioles at the A- to G-phase transition was timely correlated with a stop in the generation of new Cen2GFP structures (Fig. 1a,c,d; orange), confirming that procentriole formation occurs exclusively during A-phase.

The accumulation of centriole duplication and deuterosome markers (Cep152, Plk4, Sas6, Deup1) at the daughter centriole, the observed association of deuterosomes to its proximal part by electron microscopy and the apparent formation of Cen2GFP structures from the centrosomal region by live imaging led us to conclude that amplification proceeds from the daughter centriole^8^ (Fig. 1a, Supplementary Movie 1). To complement these previous data that were majority based on Cen2-GFP live imaging and electron-microscopy, we used a homemade Deup1 antibody to further analyze deuterosome formation in the cells both *in vitro* and *in vivo*. We classified the cells depending on the number of deuterosomes they contain, using Deup1 and Sas6 immunostainings, assuming that early amplification would correspond to small number of deuterosomes. By measuring the distances between each Deup1/Sas6 positive deuterosome and the centrosome, we quantified that the first procentriole loaded deuterosomes were observed within a radius of 1 µm from the centrosome both *in vitro* and *in vivo* (Fig. 1e,f). Then, the mean deuterosome-centrosome distance increased with the number of deuterosomes in the cell (Fig. 1e,f). Of note, Cen2GFP dynamics shows that the halos moving away from the centrosomal region can subsequently move back to it (Supplementary Movie 1), a behavior that could account for the large dispersion we observe in the centrosome-deuterosome distance (Fig. 1e,f). As previously described *in vitro*^8^, an asymmetric localization of Deup1 to the daughter centriole was observed *in vivo* during A-phase (Fig. S4). Altogether, these additional data confirm the asymmetric association of deuterosomes with the daughter centriole and strengthen the previously proposed scenario^8^ where centriole clusters organized around deuterosomes are sequentially formed within the centrosome prior to moving away in the cytoplasm.

Because two studies reported that Deup1 staining could be observed without procentriole markers^12,13^, we also decided to investigate whether this phenotype of “unloaded deuterosomes” is a reproducible step of differentiation in brain MCC. By observing Deup1 and Sas6 co-stainings in cultured cells we found that only around 20% of Deup1 positive cells possessed Deup1 signal without Sas6 (Fig. 1g,i). Within these cells, some contained only unloaded deuterosomes (“Deup1+ SAS6−”) while others a mixed population of loaded and unloaded deuterosomes (“Deup1+ SAS6+/−”). Because cells in culture can display abnormal features, we reproduced the experiments *in vivo*, in P2 mouse brain ventricles, and confirmed the *in vitro* results (Fig. 1h,i). The overlapping distribution of deuterosome number between Deup1+/SAS6− and Deup1+/SAS6+ populations (Fig. 1j) suggests that unloaded deuterosomes do not account for a systematic early stage of amplification as proposed recently^13^. In fact, the large majority of cells both *in vitro* and *in vivo* possess SAS6 loaded deuterosomes, even when very few deuterosomes are present in the cell (Figs 1i,j and S4).

During the revision of this paper, a manuscript provided data suggesting that small-unloaded deuterosomes were appearing widely distributed in the cytoplasm before procentriole formation^13^. Given the small size of the reported structures, we may have missed it because of different sensitivities of our antibodies. As we very rarely observe unloaded deuterosomes at the EM level using our serial sectioning protocol (^8^ and unpublished observations), we think correlative light and electron microscopy would be necessary to distinguish bona fide electron dense deuterosomes from putative Deup1+/Cep152+ deuterosome precursors. In addition, increasing spatio-temporal resolution of deuterosome and centriole live imaging will be needed to clarify the very early events of deuterosome formation in relation to procentriole amplification.

### Daughter-to-mother centriole conversion at the A- to G-phase transition

**Figure 2:**
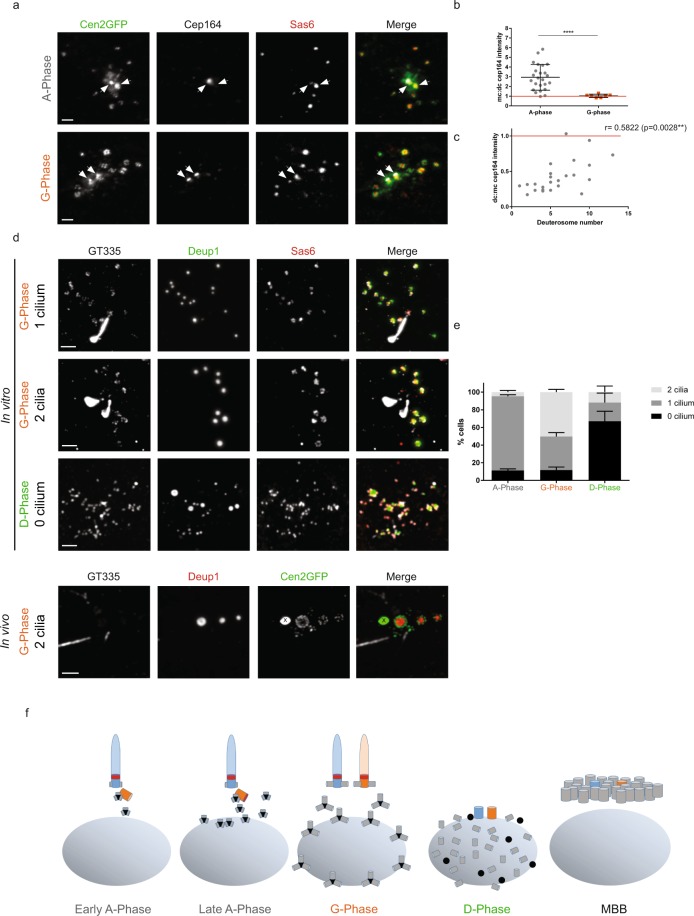
Daughter-to-mother centriole conversion at the A- to G-phase transition. (**a**) Cep164 and Sas6 immunostainings on Cen2GFP ependymal progenitors during A- and G-Phases. Arrows indicate centrosomal centrioles. (**b**) Mother:daughter centriole (mc:dc) Cep164 signal ratios in A-phase (n = 24 cells) and G-Phase (n = 7 cells). (**c**) Correlation between mc:dc Cep164 signal ratios and deuterosome numbers in the cells during A-Phase (n = 24 cells from **b**). (**d**) GT335, Deup1 and Sas6 immunostainings on ependymal progenitors during G- and D-Phases *in vitro* and G-phase *in vivo*. (**e**) Percentage of cells with 0, 1 or 2 GT335 positive cilia in A-, G- and D-phases *in vitro* (n = 210, 73 and 21 cells in A-, G- and D-phase, respectively). (**f**) Representative scheme of centrosomal centriole behaviour during centriole amplification. Nucleus is represented in gray, deuterosomes in black, procentrioles in gray, Cep164 in red, mother centriole in blue, daughter centriole in orange. « X » indicates Cen2GFP aggregate. Scale bars: 2 µm.

We next characterized centrosome behavior during the amplification process. We first found that the daughter centriole Cep164 staining evolved. The daughter centriole did not stain for Cep164 at the beginning of A-phase (Fig. 2a). However, as the A-phase progressed and the number of deuterosome increased, the daughter centriole gradually stained positive for Cep164 to finally become indistinguishable from the mother centriole during the G-phase (Fig. 2a–c). This daughter-to-mother centriole conversion was confirmed by the growth of a second primary cilium during the G-phase (Fig. 2d,e). Such bi-ciliated cells were also observed *in vivo* confirming that it is not an artifact of the culture system (Fig. 2d). These two cilia then depolymerize during the disengagement phase, consistent with the migration of the centrosome and deuterosomes to the nuclear membrane^5^, and centrosomal centrioles are no longer distinguishable from mature disengaged procentrioles (Fig. 2d,e). Altogether, these data highlight a time-correlation between parental centriole modifications and centriole amplification progression (Fig. 2f).

### Depletion of centrosomal centrioles only slightly affects centriole amplification in brain MCC

**Figure 3:**
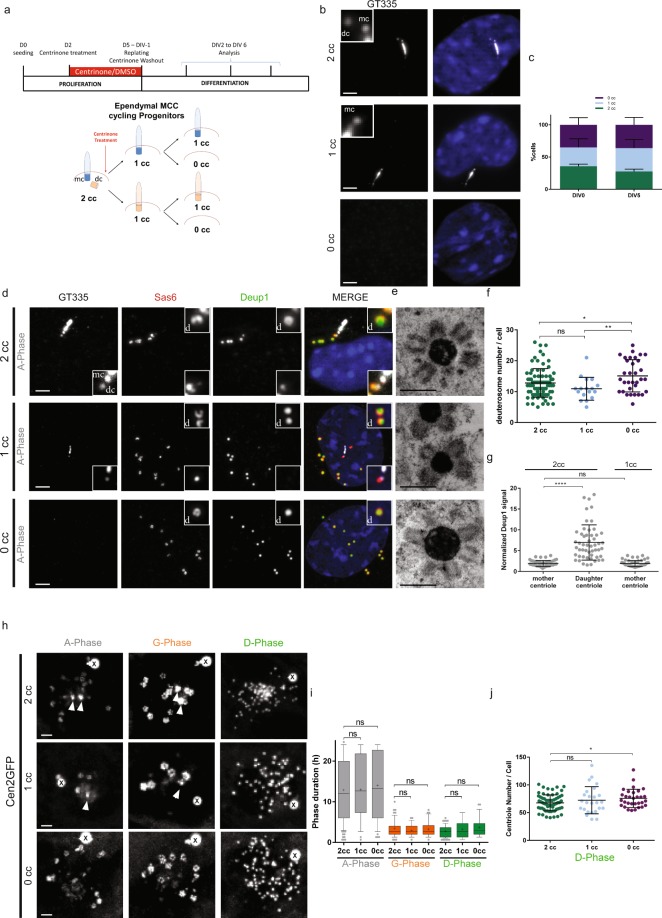
Depletion of centrosomal centrioles only slightly affects centriole amplification in brain MCC. (**a**) Experimental procedure used to deplete centrosomal centrioles in MCC cycling progenitors using centrinone. See methods. 2cc, 1cc or 0cc for 2, 1 or 0 centrosomal centriole(s), respectively. (**b**) Representative pictures of 2cc, 1cc or 0cc ependymal progenitor cells stained with GT335. (**c**) Repartition of 2cc, 1cc or 0cc cell populations at two different days of differentiation (DIV for Day *In Vitro*). Cells selected for this quantification are ependymal progenitors that have not started centriole amplification (« centrosome » stage; n = 912 and 566 cells at DIV0 and DIV5 respectively). (**d**) Representative GT335, Deup1 and Sas6 immunostainings of 2cc, 1cc or 0cc cells during A-Phase. Zoom in pictures highlight deuterosomes « d » and centrosomal centrioles (mc: mother centriole and dc: daughter centriole). (**e**) Representative EM pictures of deuterosomes in 2cc, 1cc or 0cc cells. The status of the centrosome was identified by correlative light and electron microscopy. (**f**) Quantification of deuterosome number per cell in 2cc, 1cc or 0cc cells during G-Phase. 2cc cells have been taken from DMSO and centrinone-treated cultures (n = 80, 17, 33 cells for 2cc, 1cc and 0cc, respectively). (**g**) Normalized Deup1 signal (centrosomal centriole:cytoplasmic Deup1 signal) on mother and daughter centriole in 2cc cells, or on mother centriole in 1cc cells during A-Phase (n = 52 and 54 cells for 2cc and 1cc respectively). (**h**) Sequences from time lapse experiments on 2cc, 1cc or 0cc Cen2GFP cells. Note that a Cen2GFP cloud is still present in 0cc cells. (**i**) Box (25 to 75%) and whisker (10 to 90%) plots of A- (Gray), G- (Orange), and D- (Green) phase duration in 2cc, 1cc or 0cc Cen2GFP progenitors. Lines indicate medians, and crosses indicate means. 2cc cells have been taken from DMSO and centrinone-treated cultures (in A-Phase: n = 107 cells for 2cc; n = 36 cells for 1cc; n = 49 cells for 0cc; in G-Phase: n = 73 cells for 2cc; n = 32 cells for 1cc; n = 36 cells for 0cc; in D-Phase: n = 65 cells for 2cc; n = 25 cells for 1cc; n = 28 cells for 0cc). (**j**) Final centriole number counted during D-Phase in 2cc, 1cc or 0cc Cen2GFP progenitors. Because centrosomal centrioles are no longer distinguishable from maturing procentrioles in D-Phase, quantification is performed after Cen2GFP videomicroscopy to know the orignal centrosome status of the cells. 2cc cells have been taken from DMSO and centrinone-treated cultures (n = 69 cells for 2cc; n = 25 cells for 1cc; n = 31 cells for 0cc). Arrows indicate centrosomal centrioles. « X » indicates GFP aggregates. Scale bars: 2 µm for optical microscopy, 500 nm for EM.

Because present and previous data suggested that deuterosomes and procentrioles arose from the centrosome, and more particularly from the daughter centriole^8^, we took advantage of a new drug to remove centrosomal centrioles. We used the small molecule inhibitor centrinone^14^, which inhibits Plk4 kinase activity, to deplete centrosomal centrioles from cycling MCC progenitors (Fig. 3a). By treating primary progenitor cells with centrinone for 3 days during the proliferating phase, we obtained a mixed population of cells with 2 centrosomal centrioles (2cc), 1 centrosomal centriole (1cc) or 0 centrosomal centriole (0cc) (Fig. 3a,b). Although centrinone-treated proliferating progenitors reached confluence later when compared to control (not shown), they were able to proliferate confirming that mouse cells are less sensitive than human cells to centrosome-loss for cell division^6,14–18^. This mixed population of progenitors was then washed out for centrinone and seeded at high confluence in serum free medium to trigger MCC differentiation. We obtained equivalent proportions of 2cc, 1cc and 0cc cells in differentiating cultures. These proportions did not vary between the first day of differentiation (Day *In Vitro* 0 or DIV0) and DIV5 suggesting that centriole-depleted cells are not significantly more prone to cell death (Fig. 3c). Using single-cell approaches, we first confirmed by correlative light and electron microscopy (CLEM) that Cen2GFP was suitable to assess the presence of centrosomal centrioles in the cells (Figs S1 and S2). Then, using immunostainings and CLEM, we sought to characterize centriole amplification in the 3 cell populations. Interestingly, parental centriole depletion did not block MCC progenitor capacity to form deuterosomes and procentrioles (Fig. 3d,e; Figs S1, S2 and S5). However, 0cc cells possessed slightly more deuterosomes than 1cc or 2cc cells (Fig. 3f). This is also observed in two recent studies published during the revision of the present paper, where authors treated cells with centrinone throughout proliferation and differentiation of ependymal and tracheal cell progenitors^13,19^. This suggests that centrosomal centrioles could tune deuterosome number. As previously shown^8^, Deup1 accumulation was observed at the daughter centriole in 2cc cells. Removing the daughter centriole (1cc cells) did not change the incapacity of the mother centriole to accumulate Deup1 (Fig. 3g), confirming an asymmetry in the propensity of the mother versus daughter micro-environment for Deup1 concentration and putative deuterosome formation. Next, we scored the dynamics of amplification using single cell live imaging and found that the 3 populations achieved the stereotypical phases of centriole amplification (A-, G- and D-phases) with a similar spatiotemporal pattern (Fig. 3h,i). Consistent with the increase in deuterosome number, a slight increase in the number of centrioles was observed in 0cc cells as compared to 2cc cells (Fig. 3j). Altogether our data suggest that in multiciliated cells, as previously observed in cycling cells, resident centrioles are dispensable organelles for centriole biogenesis.

### Centrosomal centriole-depleted cells form procentrioles within an acentriolar PCM cloud

**Figure 4:**
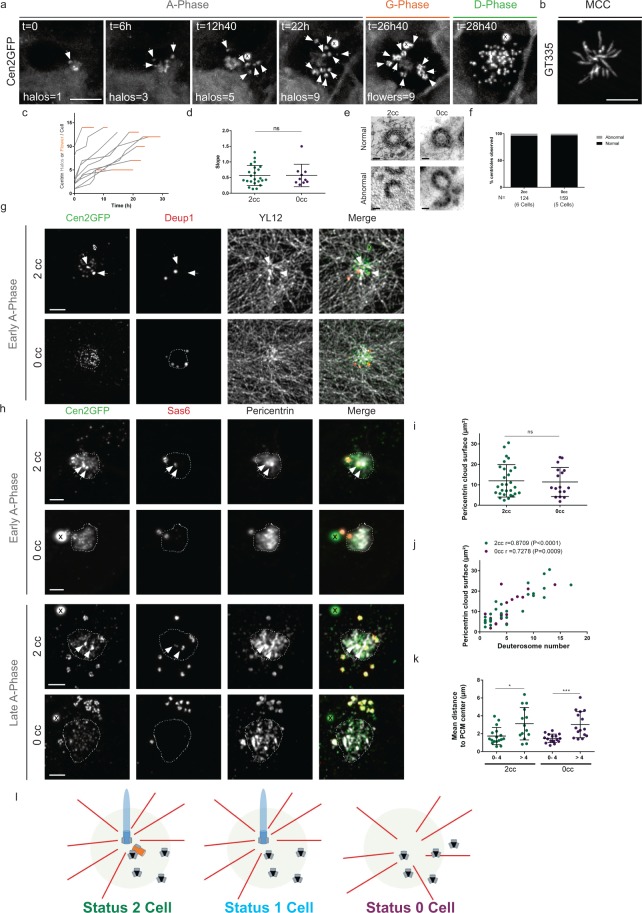
Centrosomal centriole-depleted cells form procentrioles within an acentriolar PCM cloud. (**a**) Live imaging of a Cen2GFP ependymal progenitor depleted from centrosomal centrioles undergoing the different stages (A-, G- and D-Phase) of centriole amplification. (**b**) GT335 immunostaining of a 0cc Cen2GFP ependymal MCC at the end of the time lapse experiment. (**c**) Number of centrin halos (Gray) or flowers (Orange) during time lapse experiments in 0cc Cen2GFP ependymal progenitors (Δt = 40 min, n = 10 cells). Timepoints are chosen when halos or flowers number is clearly visible. (**d**) Comparison of centrin halo formation rate between 2cc or 0cc cells during A-Phase. Each dot represents the trendline slope corresponding to a cell observed during A-Phase in Fig. 1c (2cc cells) and (**c**) (0cc cells). (**e**) Representative pictures of normal and abnormal centrioles ultrastructure in 2cc or 0cc cells observed at the multiple basal body stage. See methods. (**f**) Quantification of the integrity of centrioles ultrastructure in 2cc (6 cells observed in differentiated control cultures) and 0cc cells (5 cells observed with CLEM after videomicroscopy; See Methods). (**g**) Immunostainings showing deuterosome localization (Deup1) and the tyrosinated tubulin network (YL12) in 2cc or 0cc cells during early A-Phase. In 2cc cells, microtubule network converges on one centrosomal centriole whereas in 0cc cells, it converges on Cen2GFP cloud. Dashed line delineates Cen2GFP cloud. (**h**) Representative pictures of Pericentrin and Sas6 immunostainings during early or late A-Phase in 2cc or 0cc cells. Dashed line delineates the Pericentrin cloud. (**i**) Surface of the Pericentrin cloud in 2cc and 0cc cells during A-phase (n = 29 cells for 2cc, n = 17 cells for 0cc). (**j**) Correlation between the Pericentrin cloud surface and the number of deuterosomes in the cell in 2cc (green) or 0cc (purple) cells (n = 29 cells for 2cc, n = 17 cells for 0cc). (**k**) Mean distance of deuterosomes to PCM center depending on the number of deuterosomes in the cells for 2cc and 0cc cells (for 2cc: 0–4 deuterosomes: n = 17 cells; >4 deuterosomes: n = 14 cells. for 0cc: 0–4 deuterosomes: n = 16 cells; >4 deuterosomes: n = 15). (**l**) Model for centriole amplification in 2cc, 1cc or 0cc cells. PCM cloud is represented in light green, deuterosomes in black, procentrioles in gray, microtubules in red. Arrows indicate centrosomal centrioles. « X » indicates GFP aggregates. Scale bars: (**a**,**b**) 5 µm: (**e**) 100 nm, (**g**,**h**) 2 µm.

Finally, we focused on 0cc cells in order to decipher the precise dynamics of *de novo* centriole amplification. *De novo* centriole assembly has been induced in human cycling cells depleted from centrosomal centrioles using laser ablation^20,21^, centrinone treatment^14^ or an auxin-dependent Plk4 degradation system^18^. The supernumerary centrioles were proposed to arise all at once during S-phase^21^ either from a PCM cloud^20^ or stochastically throughout the cytosol^18^. Using live imaging on 0cc MCC progenitors, we observed that Cen2GFP halos assembled sequentially, within a normal A-phase duration (Fig. 3i), at a similar rate when compared to 2cc cells (Fig. 4a,c,d, Supplementary Movie 2), and gave rise to basal bodies growing cilia (Fig. 4b). Because these centrioles were formed without a parental template, we decided to investigate their structure. To proceed, we set up a correlative live and electron microscopy (ClivEM) assay to analyze basal body ultrastructure in cells imaged from the A-phase, where the centrosome status can be assessed. No overt ultrastructural defects were observed in basal bodies formed in centrosome-depleted cells, suggesting that parental centrioles are not necessary to provide a structural template in MCC (Fig. 4e,f).

Since we proposed previously that the sequential formation of deuterosomes and associated procentrioles was due to their sequential generation from the daughter centriole^8^, we analyzed their origin in absence of centrosomal centrioles. Parental centriole depletion by centrinone in human interphase cells has been shown to involve microtubule organization from the Golgi apparatus or from multiple cytoplasmic foci^22,23^. In MCC progenitors, the existence of a focal Cen2GFP cloud, from where the first halos were seen arising (Fig. 4a, Supplementary Movie 2), suggested that a single acentriolar MTOC was self-organizing in the absence of resident centrioles. Consistently, the Cen2GFP cloud localized at the center of convergence of the microtubule network (Figs 4g and S3a) and colocalized with a single Pericentrin^+^ Cdk5Rap2^+^ cloud (Figs 4h and S3b,c). This acentriolar MTOC did not appear connected to the Golgi apparatus (Fig. S3d). While the Pericentrin cloud surface was not modified by the status of the centrosome (Fig. 4i), its size correlated with the number of deuterosomes in both 0cc and 2cc cells (Fig. 4j). By measuring the mean distance of deuterosomes to the PCM cloud center, we revealed comparable results for 2cc and 0cc cells where the mean distance of deuterosomes to the PCM center increased with the number of deuterosomes (Fig. 4k). Altogether, this suggests that, in the presence or absence of centrosomal centrioles, deuterosomes and their procentrioles arise sequentially from a focal region, characterized by microtubule convergence and PCM accumulation (Fig. 4l).

## Discussion

In this study, we show that centrosomal centrioles are dispensable for deuterosome formation and centriole amplification in brain MCC. This is consistent with two other studies published during the revision of the present paper where the authors used either centrinone or shRNA to inhibit Plk4 function throughout the MCC differentiation program in brain or tracheal cells. Because live-imaging observations, immunocytochemistry quantifications, and EM data argued for an amplification from the daughter centriole^8^, we also assessed the centriole amplification dynamics in centrosome depleted cells. Interestingly, we found that, even in the absence of centrosomal centrioles, the procentrioles clusters seem to appear sequentially from a focal region characterized by PCM accumulation.

Although parental centrioles were found to be dispensable, the data presented here further demonstrate *in vivo* the asymmetry previously described *in vitro* between mother and daughter centrioles^8^. Deup1 structures were found associated with the daughter centriole in 60% of A-phase cells in brain ventricles. Several hypotheses can be made to explain the “a priori” discrepancy between the observed association of deuterosomes with the daughter centrioles in control cells^8^, and the fact that deuterosomes and procentrioles can form in absence of parental centrioles. First, similar to what was proposed in cycling cells, a pre-existing centriole, while dispensable, could be needed to provide a robust structural pattern^24^. Here we show that parental centriole-depleted cells form centrioles with no overt ultrastructural defects therefore suggesting that parental structures are not needed for a correct patterning of new centrioles, at least in MCC. Second, as in cycling cells, centriolar walls may serve as preferential but dispensable sites facilitating the control of nucleation events. In line with this hypothesis, parental centriole depleted cells show a slight increase in the number of deuterosomes and centrioles produced. Third, only a subset of deuterosomes/procentrioles could be nucleated on the daughter centriole, while the others are formed within the PCM. This cannot be excluded because the spatio-temporal resolution of the live imaging does not allow resolving the site of formation of each single deuterosome/procentriole. Finally, one could also argue that deuterosomes are all generated in the PCM, and then only “associate” transiently with the daughter centriole. Such connection of deuterosomes with daughter centrioles could be involved in the loading of some procentrioles generated there. In line with this latter hypothesis, SAS6 asymmetry at the daughter centriole *in vivo* is systematic^8^ but Deup1 asymmetry is observed in only 60% of the cells.

Importantly, our centrinone assay demonstrates that even in absence of the daughter centriole (“1cc cells”), the mother centriole is unable to accumulate Deup1. Since the PCM seems to be the site for deuterosome and procentriole assembly in the absence of centrosomal centrioles, mother/daughter asymmetry may reflect the existence of sub-compartments within the PCM that would be more prone to deuterosome and/or procentriole nucleation events to occur. In this line, PCM proteins are associated with deuterosomes^10^ and increasing evidence suggests that the centriolar matrix, and particularly Pericentrin, regulates centriole assembly, and not only the other way around. Overexpression of Pericentrin leads to overduplication of centrioles in human transformed cells^25^. More recently, Pericentrin was shown to be involved in centriole biogenesis and stability in *Drosophila*^26^. The increasing expression of Pericentrin during A-phase, visible by the increasing surface of the Pericentrin cloud, may be involved in the sequential formation of procentrioles we observed, even in the absence of centrosomal centrioles. Depleting pericentrin and driving the daughter centriole away from the mother centriole^27^ may allow to test the association of PCM with the daughter centriole and their respective contribution in MCC centriole amplification.

In this study, we also highlighted an atypical centrosome behavior, correlated in space and time to the progression of procentriole amplification in brain MCC. While procentriole clusters during A-phase seems to arise from the centrosomal region, the arrest in cluster formation is marked by procentriole growth at the A- to G- transition and correlates with the maturation of the centrosomal daughter centriole. Concurrent maturation of the young centrosomal centriole and the procentrioles is also seen in cycling cells and was shown to be Plk1 dependent^28^. Since Plk1 regulates the A- to G- transition in brain MCC progenitors^5^, it is tempting to speculate that it also drives the concurrent maturation of the two generations of centrioles in these differentiating cells. Interestingly, such maturation of the daughter centriole is also observed in MCC induced from primary fibroblasts^12^. The causality relationships between daughter centriole maturation, procentriole growth and amplification arrest remains to be determined.

## Methods

### Animals

All animal studies were performed in accordance with the guidelines of the European Community and French Ministry of Agriculture and were approved by the Direction départementale de la protection des populations de Paris (Approval number APAFIS#9343-201702211706561 v7). The mice used in this study have already been described and include: OF1 (Oncins France 1, Charles River Laboratories); Cen2GFP (CB6-Tg(CAG-EGFP/CETN2)3-4Jgg/J, The Jackson Laboratory). Videomicroscopy experiments were performed on cells from homozygous Cen2GFP mice. Other experiments were performed in parallel using OF1 or homozygous Cen2GFP mice; no differences between these two strains were observed regarding ependymal differentiation, amplification stages, centrosome asymmetry or number of deuterosomes and centrioles.

### Brain dissections

Whole mounts of developing ventricular walls were prepared from P2-P4 adult mice as previously described^11^.

### Primary ependymal cell cultures and centrinone treatment

Ependymal cell culture has been described previously^8,11^. Briefly, newborn mice (P0–P2) were killed by decapitation. The brains were dissected in Hank’s solution (10% HBSS, 5% HEPES, 5% sodium bicarbonate, 1% penicillin/streptomycin (P/S) in pure water) and the extracted ventricular walls were cut manually into pieces, followed by enzymatic digestion (DMEM glutamax, 33% papain (Worthington 3126), 17% DNase at 10 mg ml^−1^, 42% cysteine at 12 mg ml^−1^) for 45 min at 37 °C in a humidified 5% CO_2_ incubator. Digestion was stopped by addition of a solution of trypsin inhibitors (Leibovitz Medium L15, 10% ovomucoid at 1 mg ml^−1^, 2% DNase at 10 mg ml^−1^). The cells were then washed in L15 and resuspended in DMEM glutamax supplemented with 10% fetal bovine serum (FBS) and 1% P/S in a Poly-l-lysine (PLL)-coated flask. Ependymal progenitors proliferated for 5 days until confluence followed by shaking (250 rpm) overnight. Pure confluent astroglial monolayers were replated at a density of 7 × 10^4^ cells per cm^2^ (corresponding to days *in vitro* (DIV) −1) in DMEM glutamax, 10% FBS, 1% P/S on PLL-coated coverslides for immunocytochemistry experiments, Lab-Tek chambered coverglasses (Thermo Fisher Scientific) for time-lapse experiments or glass-bottomed dishes with imprinted 50 µm relocation grids (Ibidi, catalogue no. 81148, Biovalley) for correlative Light/EM and maintained overnight. The medium was then replaced by serum-free DMEM glutamax 1% P/S, to trigger ependymal differentiation gradually *in vitro* (DIV 0). Centrinone was added on day2 of the proliferation (D2) phase at a final concentration of 0.6 µM. Centrinone was washed out 3 times with PBS on day 5 (D5) of the proliferation phase just before trypsinisation and replating at high confluence for MCC differenciation.

### Immunostainings

Lateral brain ventricles were first pre-permeabilized in 0.2% Triton X-100 BRB medium (80 mM PIPES, 1 mM MgCl_2_, 1 mM EGTA) for 2 min before fixation. Brain or cell cultures (between DIV2 and DIV6) were fixed in methanol at −20 °C for 10 min. Samples were pre-blocked in 1 × PBS with 0.2% Triton X-100 and 10% FBS before incubation with primary and secondary antibodies. Tissues or cells were counterstained with DAPI (10 μg ml^−1^, Sigma) and mounted in Fluoromount (Southern Biotech). The following antibodies were used: rabbit anti-Cep164 (1:750)^29^; rabbit anti-Deup1 (1:2000) (homemade, raised against the peptide TKLKQSRHI); mouse IgG1 anti-GT335 (1:500, Adipogen); mouse IgG2b anti-Sas6 (1:750, Santa Cruz); rabbit anti-Pericentrin (1:2000, covance); rat anti-YL12 (1:500, abcam) and species-specific Alexa Fluor secondary antibodies (1:400, Invitrogen).

### Microscopy

#### Epifluorescence microscopy

Fixed cells and whole-mount ventricles were examined with an upright epifluorescence microscope (Zeiss Axio Observer.Z1) equipped with an Apochromat ×63 (NA 1.4) oil-immersion objective and a Zeiss Apotome with an H/D grid. Images were acquired using Zen with 240-nm z-steps.

#### Confocal super-resolution microscopy

Confocal image stacks were collected with a 63 x/1.4 Oil objective on an inverted LSM 880 Airyscan Zeiss microscope with 440, 515, 560 and 633 laser lines.

#### Videomicroscopy

Videomicroscopy has been described previously^8^. Briefly, cultured cells between DIV2 and DIV6 were filmed *in vitro* using an inverted spinning disk Nikon Ti PFS microscope equipped with oil-immersion ×63 (NA 1.32) and ×100 (NA 1.4) objectives, an Evolve EMCCD Camera (Photometrics), dpss laser (491 nm, 25% intensity, 70–100 ms exposition), appropriate filter sets for DAPI/FITC/TRITC, a motorized scanning deck and an incubation chamber (37 °C; 5% CO_2_; 80% humidity). Images were acquired with Metamorph Nx with 40 min time intervals. Image stacks were recorded with a z-step of 0.7 µm. After film acquisition, cells were then fixed for 5 min with 0.5% paraformaldehyde (PFA) using a Pasteur pipette without moving the chambered coverglass, and GT335 primary and secondary antibodies were added together for 25 min, in medium supplemented with FBS (10%) before the final images were acquired.

#### Correlative light and electron microscopy (CLEM)

CLEM on ependymal *in vitro* culture cells has been described elsewhere^8^. Primary Cen2GFP ependymal progenitors were grown in 0.17-mm thick glass dishes with imprinted 50 µm relocation grids (Ibidi). At 3–6 days *in vitro* (DIV 3–6), cells were fixed with 4% PFA for 10 min and ependymal progenitors undergoing A-phase or G-Phase were imaged for Cen2GFP and DAPI, in PBS, with upright epifluorescence microscope (Zeiss Axio Observer.Z1). Coordinates on the relocation grid of the cells of interest were recorded. Cells were then treated for transmission electron microscopy. Briefly, culture cells were treated with 1% OsO4, washed and progressively dehydrated. The samples were then incubated in 1% uranyl acetate in 70% methanol, before final dehydration, pre-impregnation with ethanol/epon (2/1, 1/1, 1/2) and impregnation with epon resin. After mounting in epon blocks for 48h at 60 °C to ensure polymerization, resin blocks were detached from the glass dish by several baths in liquid nitrogen. Using the grid pattern imprinted in the resin, 50 serial ultra-thin 70-nm sections of the squares of interest were cut on an ultramicrotome (Ultracut EM UC6, Leica) and transferred onto formvar-coated EM grids (0.4 × 2 mm slot). The central position of the square of interest and DAPI staining were used to relocate and image the cell of interest using a Philips Technai 12 transmission electron microscope. Four different 0cc cells and two 1cc cells have been analyzed to verify our ability to determine centrosomal status from Cen2-GFP signal.

Centrioles ultrastructure was determined by Correlative Live and Electron Microscopy (CLivEM) after videomicroscopy. Briefly, Cen2-GFP cells were plated in 0.17-mm thick glass dishes with imprinted 50 µm relocation grids (Ibidi). Cells with 2cc or 0cc undergoing centriole amplification were then filmed with an inverted spinning disk Nikon Ti PFS microscope until they reached multiple basal body stage and then proccessed for CLEM imaging such as described before^8^.

### Quantification and statistics

Rates of Cen2GFP structures appearance: we quantified the number of centrin halos or flowers at different times and computed the slopes given by a linear regression. Distance between deuterosomes and the centrosome: we measured the distance between the deuterosome center of mass and the middle of the segment drawn between the two centrosomal centrioles. The mean distance was then calculated within each single cell. Mean distance of deuterosomes to Pericentrin cloud center: a region of interest corresponding to the Pericentrin cloud was manually drawn and centroid coordinates were then used as the PCM center. The same protocol was used for Pericentrin cloud surface measurements. Centriole final number: it was always determined using the Cen2-GFP signal of D-Phase cells monitored by videomicroscopy from A-phase in order to identify the initial status of the centrosome. Because centriole number (D-phase) or deuterosome number (G-Phase) of 2cc cells from DMSO and centrinone-treated cell were not significantly different, we decided to pool 2cc-DMSO and 2cc-centrinone data sets in the quantifications.

Fluorescence signal intensities were quantified using Image J. All graphs and statistical analyses were obtained using GraphPad Prism software. Data were obtained from at least three independent experiments and the results presented as the mean ± s.d. Non-parametric two-tailed Mann–Whitney U-tests were used to compare groups of data. Pearson’s correlation coefficient was calculated to determine the strength of the relationship between two variables. For P-Values: ns: P > 0.05; *: P ≤ 0.05; **: P ≤ 0.01; ***: P ≤ 0.001; ****: P ≤ 0.0001.

A previous version of the manuscript has been uploaded on BioRxiv as a preprint and can be found under the following link: https://www.biorxiv.org/content/10.1101/503730v1.

## Supplementary information

Supplementary movie 1

Supplementary movie 2

Supplementary information

## Data Availability

All data generated or analyzed during this study are included in this published article (and its Supplementary Information files).
