# Supplementary material for: Dynamics of centriole amplification in centrosome-depleted brain multiciliated progenitors
Source: Sci Rep. 2019 Sep 10;9:13060. doi: 10.1038/s41598-019-49416-2 (PMC6736942; doi:10.1038/s41598-019-49416-2)
Supplement: Supplementary file 3 — Supplementary information [file 41598_2019_49416_MOESM3_ESM.pdf]

Dynamics of centriole amplification in centrosome-depleted brain multiciliated progenitors

Olivier Mercey[1, 2, 3*], Adel Al Jord[4], Philippe Rostaing[1, 2, 3], Alexia Mahuzier[1, 2, 3], Aurélien Fortoul[1, 2, 3], Amélie-Rose Boudjema[1, 2, 3], Marion Faucourt[1, 2, 3], Nathalie Spassky[1, 2, 3], Alice Meunier[1, 2, 3*]

[1]Institut de Biologie de l'École Normale Supérieure (IBENS), Paris Sciences et Lettres (PSL) Research University, Paris F-75005, France.
[2]CNRS, UMR 8197, Paris F-75005, France.
[3]INSERM, U1024, Paris F-75005, France.
[4]Center for Interdisciplinary Research in Biology (CIRB), Collège de France, CNRS 7241 INSERM U1050; PSL Research University, Paris 75005, France

*Corresponding authors: Olivier Mercey (mercey@biologie.ens.fr) and Alice Meunier (alice.meunier@ens.fr)

Supplementary Figure 1 : Characterization of a daughter centriole-depleted cell

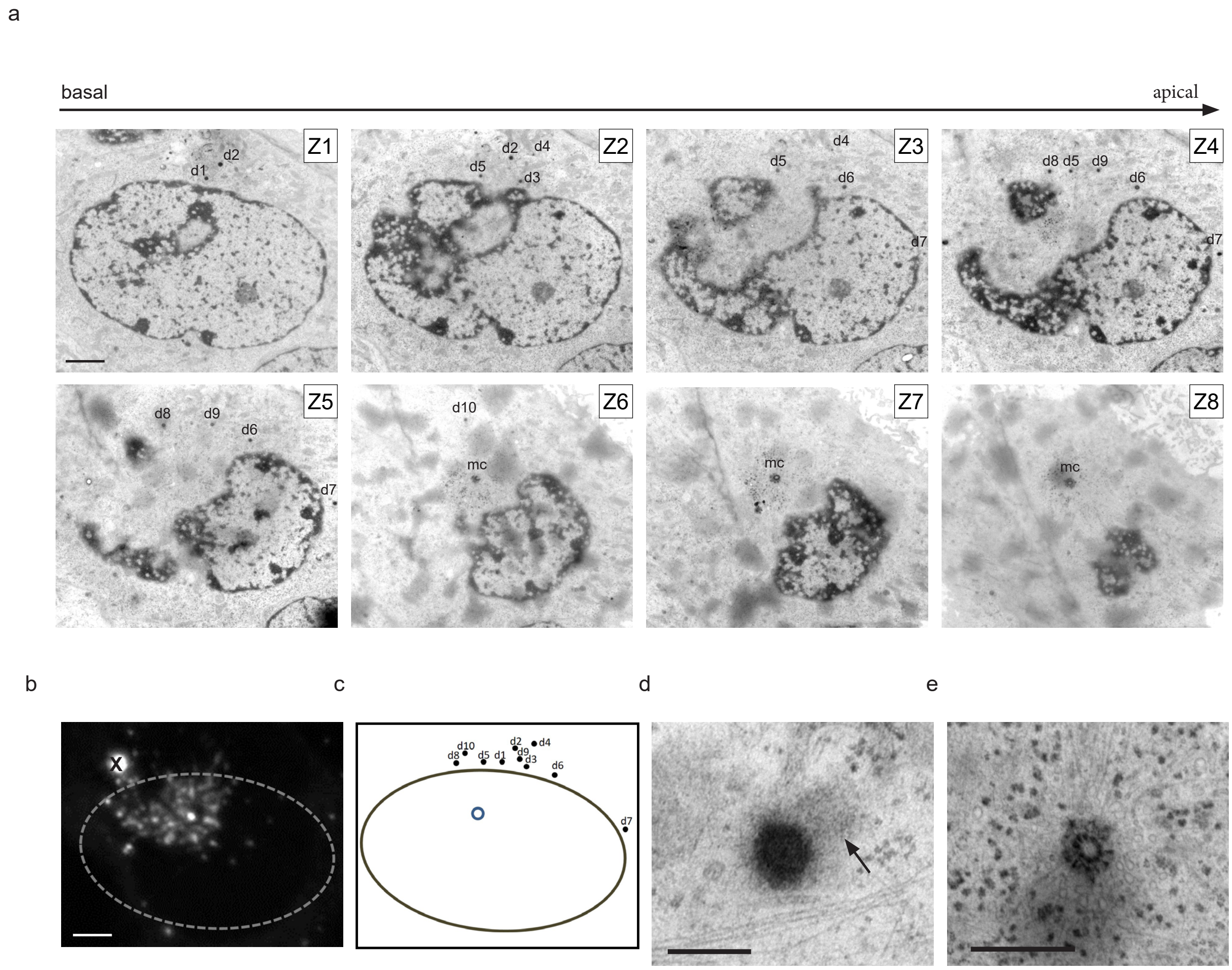

Supplementary Figure 1

Characterization of a daughter centriole-depleted cell

a. EM serial pictures of a daughter centriole-depleted cell (1cc). 1cc cell is first selected using Cen2GFP signal (**b**). First representative picture (Z1) corresponds to the first ultrathin section (70nm) where deuterosomes « d » are observed. Deuterosomes are then counted in order of appearance. mc: mother centriole. Scale bar: 2µm. **b**. Cen2GFP signal of the daughter centriole-depleted cell observed in EM. Dashed line represents the nucleus. « X » indicates Cen2GFP aggregate. Scale bar = 2µm. **c**. Schematic representation of the cell observed in CLEM. Black dots represent deuterosomes, Solid line indicates nucleus and blue circle represents mother centriole. **d**. Zoom-in of a deuterosome with one growing procentriole. Arrow indicates the procentriole. Scale bar: 200nm. **e**. Zoom-in on the mother centriole surrounded by aggregates and organizing microtubules. Scale bar: 1µm.

Supplementary Figure 2 : Characterization of a centrosomal centriole-depleted cell

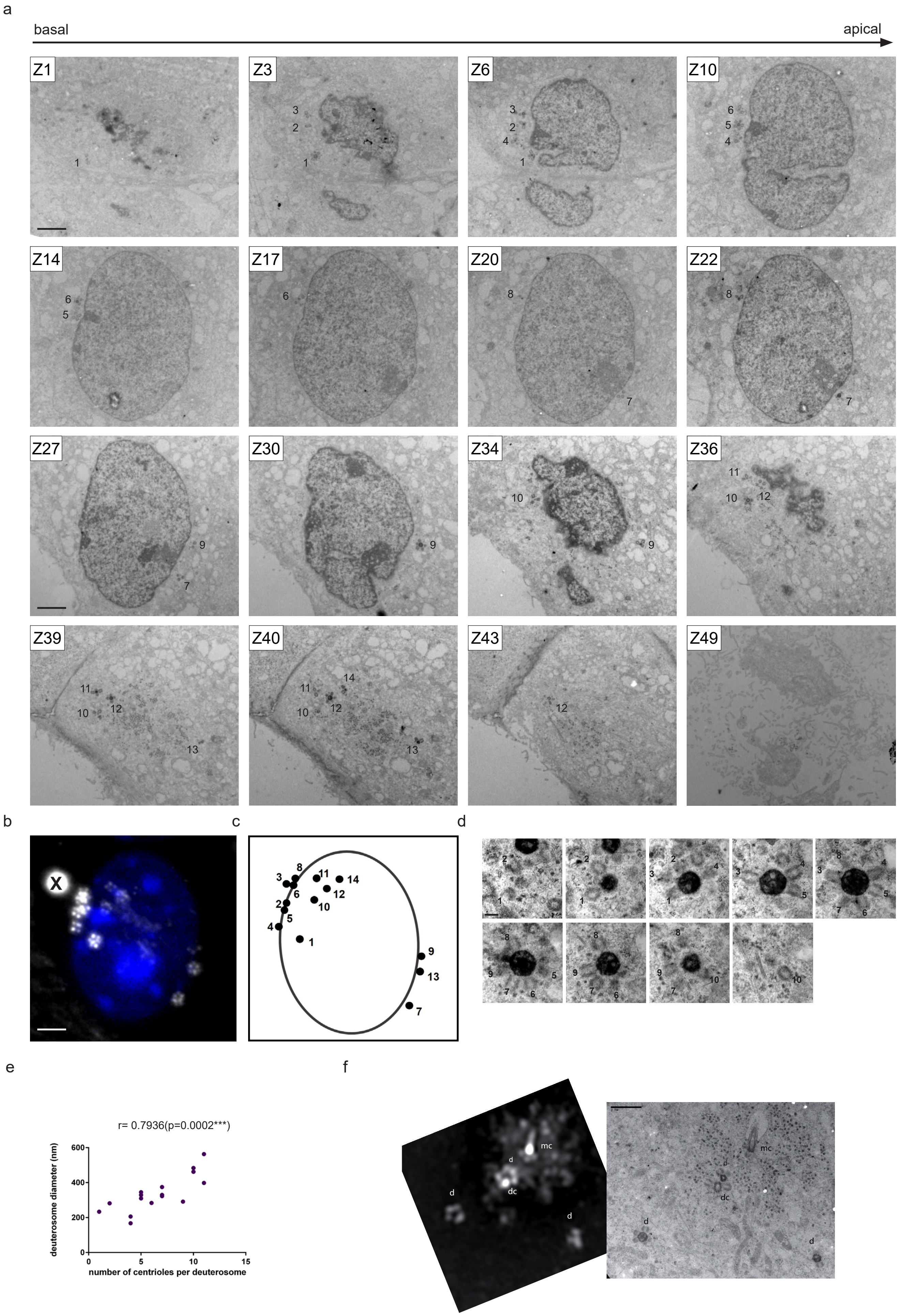

Supplementary Figure 2

Characterization of a centrosomal centriole-depleted cell

a. EM serial pictures of a parental centriole-depleted cell (0cc). 0cc cell is first selected using Cen2GFP signal (b). First section (Z1) corresponds to the first Ultrathin section (70nm) where deuterosomes « d » are observed. Deuterosomes are then counted in order of appearance. Sections are selected to have a global vision of all deuterosomes but keep their original number. Scale bar: 2µm. **b**. Cen2GFP signal of the parental centriole-depleted cell observed in EM. « X » indicates Cen2GFP aggregate. Scale bar: 2µm. **c**. Schematic representation of the cell observed in CLEM. Black dots represent deuterosomes, Solid line indicates nucleus. **d**. Serial pictures of a deuterosome from a 0cc cell. Numbers correspond to procentrioles. Scale bar = 200nm. **e**. Correlation between deuterosome diameter and the number of centrioles. Diameter of deuterosomes is calculated on the Ultrathin section where deuterosome surface is the largest. Number of procentrioles is calculated in the same way as in **d. f.** CLEM of a 2cc cells. On the left, Cen2GFP signal of the cell. On the right, one serial section where the two parental centrioles and deuterosomes can be observed. mc: mother centriole. dc: daughter centriole. d: deuterosome. Scale bar: 1µm.

Supplementary Figure 3 : Microenvironment of centriole amplification in centrosomal centriole-depleted cells

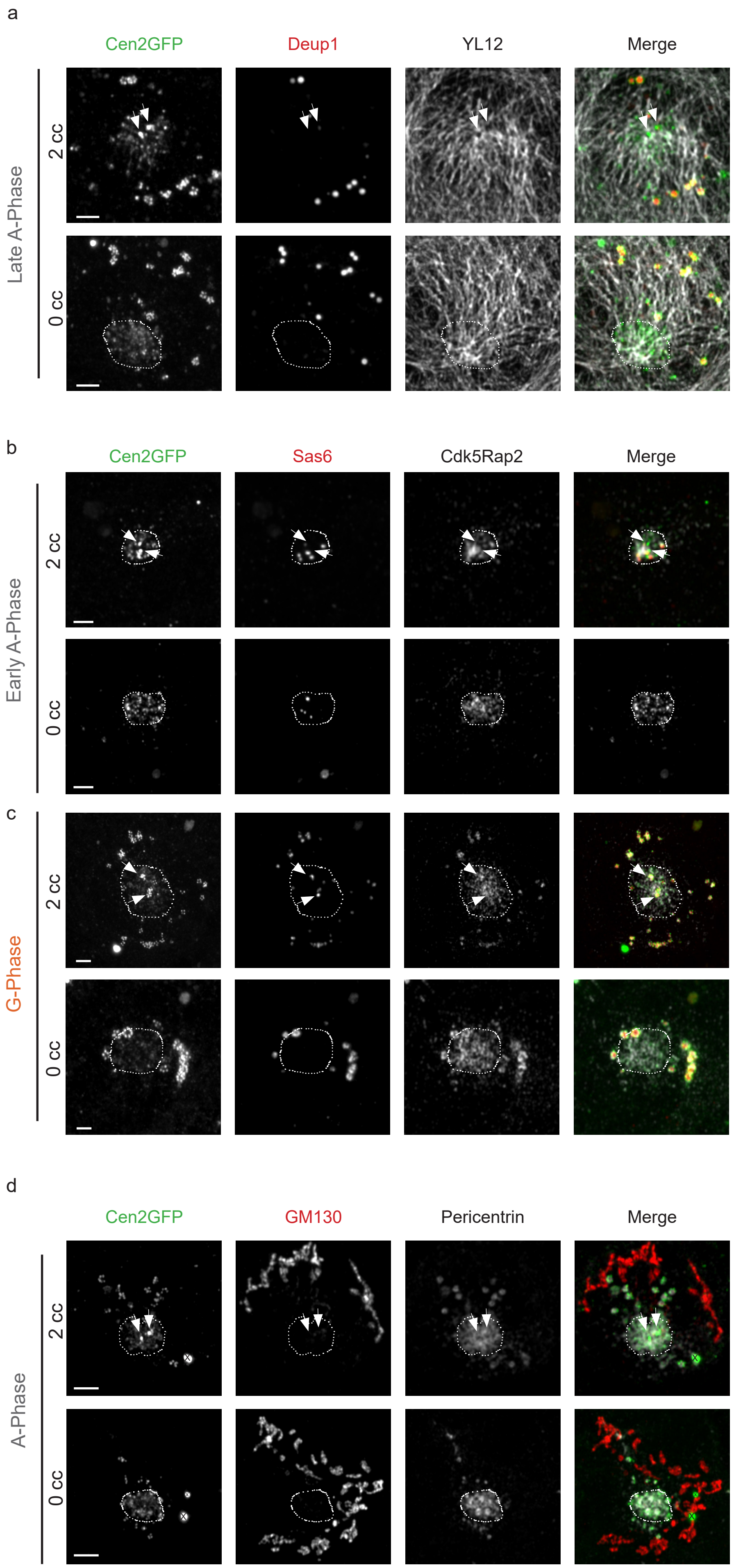

Supplementary Figure 3

Microenvironment of centriole amplification in centrosomal centriole-depleted cells

a. Immunostainings of the tyrosinated microtubule network (YL12) and deuterosomes (Deup1) in 2cc or 0cc cells during Late A-Phase. As for early A-Phase (**Fig 4e**), in 2cc cells, microtubule network converges on one centrosomal centriole whereas in 0cc cells, it converges on the Cen2GFP cloud, where the PCM is enriched. Dashed line delineates Cen2GFP cloud. **b**, **c**. Immunostainings of the PCM protein Cdk5rap2 and Sas6 during A-Phase **(b)** or G-phase **(c)** in 2cc and 0cc Cen2GFP progenitors. Dashed line delineates the PCM cloud. **d**. Immunostainings of the cis-Golgi (GM130) and Pericentrin on 2cc or 0cc Cen2GFP progenitors during A-phase. Dashed line delineates the PCM cloud. Arrows indicate centrosomal centrioles. « X » indicates Cen2GFP aggregate. Scale bar: 2µm.

Supplementary Figure 4 : *In vivo* characterization of deup1 asymmetry on centrosomal centrioles during A-Phase

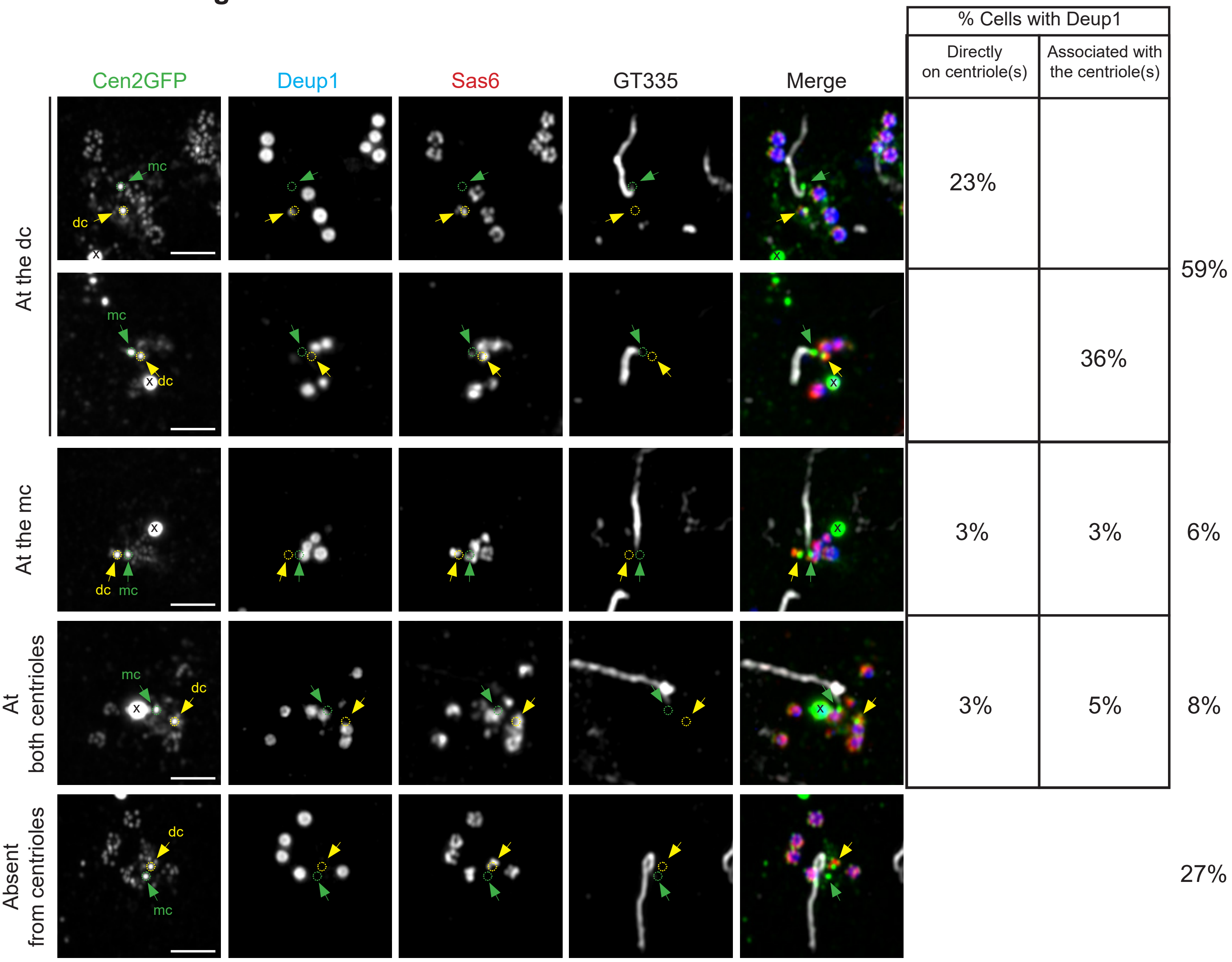

Supplementary Figure 4

***In vivo* characterization of Deup1 asymmetry on centrosomal centrioles during A-phase**

Super-resolution z-projection images and quantification of *in vivo* Cen2GFP ependymal progenitors stained for Deup1, Sas6 and GT335 during A-phase. Association of Deup1 with centrosomal centrioles is assessed by the simultaneous and close presence of Deup1 and Cen2GFP signals on the same z picture. A-phase identification is based on the Cen2GFP halo signal. Percentages are calculated from 329 cells imaged on the brain lateral ventricular walls from 5 different animals at post-natal day 2 and 4. Scale bar: 2µm.

Supplementary Figure 5 : Characterization of *in vitro* differentiation in DMSO or centrinone treated cells

a

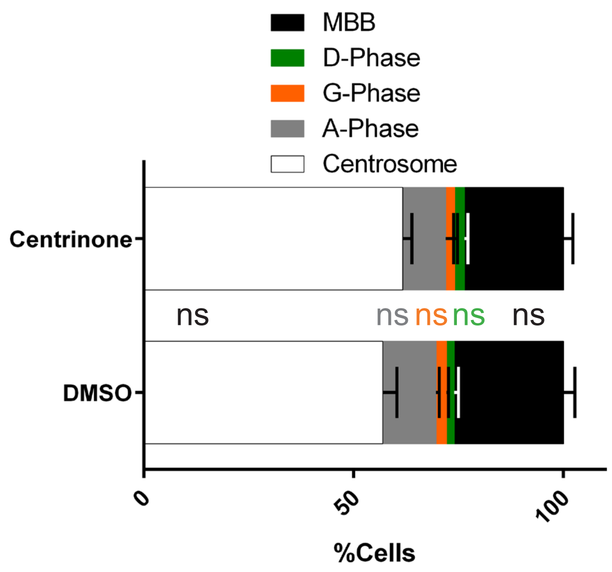

Supplementary Figure 5

Characterization of *in vitro* differentiation of DMSO or centrinone-treated cells

a. Proportions of cells in the different stages of differentiation depending on drug treatment (DMSO or Centrinone) observed between DIV2 and DIV6 from 4 different cell cultures.

Supplementary Movie 1

Live imaging of centriole amplification in a 2cc Cen2GFP progenitor

Time-lapse of the centriole amplification dynamic of the 2cc Cen2-GFP progenitor depicted in Fig 1a (63X magnification, Δt=40 minutes). « X » indicates Cen2GFP aggregate. Green arrows indicate centrosomal centrioles. Arrowheads indicate centrin halo or flowers. Note that the number of centrin halos or flowers has been measured in 3D. Scale bar: 5 µm.

Supplementary Movie 2

Live imaging of centriole amplification in a 0cc Cen2GFP progenitor

Time-lapse of the centriole amplification dynamic of the 0cc Cen2-GFP progenitor from Fig 4a (63X magnification, Δt=40 minutes). « X » indicates Cen2GFP aggregate. Arrowheads indicate centrin halo or flowers. Note that the number of centrin halos or flowers has been measured in 3D. Scale bar: 5 µm.
